# MiR-1224-5p attenuates polycystic ovary syndrome through inhibiting NOD-like receptor protein 3 inflammasome activation via targeting Forkhead box O 1

**DOI:** 10.1080/21655979.2021.1987125

**Published:** 2021-10-21

**Authors:** Yan Li, Nianling Yao, Yan Gao, Yunping Wang, Lu Bai, Jia Xu, Haixu Wang

**Affiliations:** Department of Obstetrics and Gynecology, the First Affiliated Hospital of The Fourth Military Medical University, Xi’an, Shaanxi 710032, People’s Republic of China

**Keywords:** Polycystic ovary syndrome, miR-1224-5p, human ovarian granulosa cells, NLRP3 inflammasome, FOXO1

## Abstract

Polycystic ovary syndrome (PCOS) is a common endocrine disorder that poses a great threat to women's health. MiR-1224-5p is downregulated in the follicular fluid of patients with PCOS, but its role remains largely unknown. In this study, mice were treated with dehydroepiandrosterone (DHEA) to establish an *in vivo* model of PCOS. We found that enhanced activation of NLRP3 inflammasome was accompanied by downregulation of miR-1224-5p in ovarian tissue of PCOS mice. The effect of miR-1224-5p was further explored in TNF-α-treated human granulosa-like tumor (KGN) cells. Upregulation of miR-1224-5p suppressed TNF-α-induced secretion of DHEA and testosterone. MiR-1224-5p attenuated TNF-α-induced inflammation by inhibiting NLRP3 inflammasome activation, IL-1β synthesis, and nuclear factor kappa B (NF-κB) p65 nuclear translocation. Notably, miR-1224-5p decreased the expression of Forkhead box O 1 (FOXO1) and its downstream gene thioredoxin interaction protein (TXNIP). Luciferase reporter assay confirmed FOXO1 as a target of miR-1224-5p. Upregulation of FOXO1 abolished miR-1224-5p-induced activation of NLRP3 inflammasome, demonstrating that miR-1224-5p might inhibit NLRP3 inflammasome activation through regulating FOXO1. This study provided novel insights into the pathogenesis of PCOS and suggested that miR-1224-5p might be a promising target for treating PCOS.

## Introduction

Polycystic ovary syndrome (PCOS) is a common endocrine disorder with high morbidity in women of reproductive age, which is clinically characterized by polycystic ovaries, hyperandrogenism and ovulatory abnormality [1]. Women who suffered from PCOS are always accompanied with increased risks of metabolic dysfunction, diabetes and cardiovascular diseases, which pose great threats to their health [2]. Androgen excess, abnormality of gonadotropin and insulin resistance are believed to play dominant roles in the development of PCOS [3,4]. However, the pathogenesis of PCOS is still not completely understood. Current clinical medication for treating PCOS includes oral contraceptives, antiandrogens, glucocorticoids and insulin-sensitizing agents (such as metformin) [5]. The underlying cellular mechanism of PCOS has not been fully elucidated yet and needs further exploration. A better understanding of its pathogenesis could contribute to the development of novel potential therapeutic targets for PCOS.

Chronic low-grade inflammation has been identified as a critical contributor to the pathogenesis of PCOS, which aggravates the metabolic and ovarian dysfunction. In the previous research, several inflammatory cytokines were found to be abnormally elevated in women with PCOS, such as tumor necrosis factor (TNF)-α and interleukin (IL)-6 [6]. These cytokines directly or indirectly affect the secretion of androgens and the progression of PCOS. NOD-like receptor protein 3 (NLRP3) inflammasome is an important mediator of host immune response, which consists of NLRP3, apoptosis-associated speck-like protein containing a C-terminal caspase recruitment domain (ASC) and caspase-1 [7]. NLRP3 inflammasome is activated in various inflammatory and metabolic diseases, such as type 2 diabetes [8]. As far as we know, NLRP3 inflammasome is expressed in ovarian granulosa cells (GCs) and can be abnormally activated under pathological conditions or different stimulus [9]. Exposure to hyperandrogen activated the NLRP3 inflammasome and derived ovarian dysfunction and fibrosis in mice [10]. High glucose induced NLRP3 expression in mouse GCs [11]. Non-esterified fatty acid induced NLRP3 inflammasome activation in GCs [12]. Notably, compared with normal controls, NLRP3 inflammasome was formatted/activated in ovarian GCs of PCOS patients and in KGN cells stimulated by LPS or follicular fluid from PCOS patients [13]. Although the activation of NLRP3 inflammasome is detected in patients with PCOS [14], the underlying regulatory mechanism remains largely unknown.

Forkhead box O1 (FOXO1) is a transcription factor that mediates the expression of thioredoxin interaction protein (TXNIP) and induces the generation of reactive oxygen species (ROS), which further activates NLRP3 inflammasome [15]. It is reported that TXNIP is greatly upregulated in the serum and granulosa cells of patients with PCOS [16,17]. Thus, FOXO1 may play a critical role in the pathogenesis of PCOS through regulating the expression of TXNIP and the activation of NLRP3 inflammasome.

MicroRNAs (miRNAs) are a class of small non-coding RNA molecules with the length of ~20–23 nucleotides, which function to regulate gene expression by targeting the 3ʹ untranslated regions (UTRs) of mRNAs [18]. By repressing the translation or promoting the degradation of target mRNAs, miRNAs play essential regulatory roles in cellular physiology of migration, proliferation and apoptosis. Dysfunction of miRNAs is tightly associated with the development of many human diseases, including cancers [19], neurodegenerative diseases [20], as well as metabolic disorders [21]. Recently, several miRNAs are documented to be abnormally expressed in patients with PCOS [22]. MiR-1224-5p was found to be dramatically downregulated in the follicular fluid of patients with PCOS [23]. However, the potential role of miR-1224-5p in the development of PCOS has not been investigated yet.

Furthermore, FOXO1 is predicted to be a potential target gene of miR-1224-5p. Considering the above findings, we hypothesized that downregulation of miR-1224-5p might contribute to the development and progression of PCOS through the regulation of FOXO1. In the present study, we aim to investigate the role of miR-1224-5p and FOXO1 in dehydroepiandrosterone (DHEA)-induced mouse model of PCOS and TNF-α-treated human granulosa-like tumor (KGN) cells. Moreover, the regulatory relationship between miR-1224-5p and FOXO1 was studied *in vitro*.

## Materials and methods

### Mouse model of PCOS

Female C57BL/6 mice aged 3 weeks were adaptively raised in a room under 12 h light/dark cycles for 1 week and then divided into two groups (Control and DHEA). A mouse model of polycystic ovary syndrome (PCOS) induced by DHEA was established in this study, according to previous studies [24–26]. Briefly, mice in DHEA group were subcutaneously injected with 60 mg/kg DHEA (Aladdin regents, Shanghai, China) in the nape back per day for 3 weeks, while the mice in Control group were normally raised. The fasting glucose and fasting insulin levels were determined 3 weeks later, and the homeostasis model assessment of insulin resistance (HOMA-IR) was conducted according to a reported method [27]. Total 36 mice were used for modeling, resulting in 27 mice with HOMA-IR index >2.8 after the injection. These mice were considered as the successful mouse model of PCOS, and 24 mice were used for further study. Among them, six mice were used for histology, six mice were used for real-time PCR (one ovary) and Western blot (the other one), and the rest of the mice for ELISA assays. The animal experiments were approved by the Ethics Committee of Xijing Hospital, Air Force Medical University.

### Hematoxylin and eosin (H&E) staining

H&E staining was performed to assess the pathological changes in the ovarian tissue of mice [28]. The ovarian tissue was dehydrated in gradient ethanol and embedded in paraffin. After being cut into 5-μm slices, the tissue samples were stained with hematoxylin (Solarbio, Beijing, China) solution and eosin (Sangon Biotech, Shanghai, China) solution. The samples were observed under a microscope (Olympus, Tokyo, Japan) at a magnification of 40× and 400 × .

### Serum analysis

The serum levels of luteinizing hormone (LH), follicle-stimulating hormone (FSH) and estradiol were determined by an LH ELISA kit (Fine Biotech, Wuhan, China), an FSH detection kit (USCN life science, Wuhan, China) and an estradiol ELISA kit (Fine Biotech) following manufacturer’s instruction, respectively.

### Real-time PCR

The relative mRNA expression of miR-1224-5p, NLRP3, ASC, FOXO1 and TXNIP was quantified by real-time PCR [29,30]. Total RNA was obtained from the ovarian tissue of mice or KGN cells by an RNA extraction kit (Tiangen Biotech, Beijing, China). The concentration was quantified by a UV spectrophotometer (Thermo Scientific, Rockford, IL, USA). The RNA sample was then reversibly transcribed into cDNA in a PCR system (Bioneer Corporation, Daejeon, Korea). Quantitative real-time analysis was conducted in the PCR system using the cDNA, SYBR Green (Solarbio Science & Technology), primers (Genscript, Nanjing, China) and 2× Taq PCR Master Mix (Tiangen Biotech). Data were analyzed by 2^−ΔΔCt^ formula and normalized to U6 or GAPDH. The sequences of primers are shown in Table 1.

**Table 1.** The sequences of primers

Gene	Forward primer (5ʹ-3ʹ)	Reverse primer (5ʹ-3ʹ)
Mus musculus-miR-1224-5p	GTGAGGACTGGGGAGGTG	GTGCAGGGTCCGAGGTATTC
Homo sapiens-miR-1224-5p	GTGAGGACTCGGGAGGTG	GTGCAGGGTCCGAGGTATTC
NLRP3	TTCGGAGATTGTGGTTGGG	AGGGCGTTGTCACTCAGGT
ASC	TGGATGCTCTGTACGGGAAGGT	TGTTGCTGGGAAGGAGCCTC
FOXO1	GTCCTACGCCGACCTCATC	TTGCTGTCACCCTTATCCTTG
TXNIP	GTCATCAGTCAGAGGCAATCA	AGGAACGCTAACATAGATCAGTAA

### Western blotting analysis

Western blotting was carried out as described previously with a minor modification [31,32]. Total protein was extracted from ovarian tissue or KGN cells using RIPA buffer (Solarbio Science & Technology) and centrifuged at 10,000 g for 5 min to obtain the supernatant. Nuclear and cytoplasmic protein of KGN cells was extracted using the commercial kit (Solarbio Science & Technology). Briefly, cells were treated with the cytoplasmic protein extraction reagent for 10 min in an ice bath, followed by centrifugation at 12,000 g for 10 min. The supernatant was collected as the cytoplasmic protein extracts. The precipitation was further treated with the nuclear protein extraction reagent and centrifuged as described above, and then obtained with nuclear protein extracts. The protein samples were quantified by a BCA protein assay kit (Solarbio Science & Technology). After quantification, the samples were subjected to SDS-PAGE at 80 V for 2.5 h using 5% concentration gel and 8%–15% separation gel and transferred to PVDF membranes (Millipore, Billerica, MA, USA). After that, the PVDF membranes were blocked in 5% nonfat milk and incubated with primary antibodies for NLRP3 (1:500 dilution; Cell Signaling Technology, Danvers, MA, USA), ASC (1:1000 dilution; Abclonal Technology, Wuhan, China), pro-caspase-1/cleaved caspase-1 (1:1000 dilution; Abcam, Cambridge, UK), FOXO1 (1:1000 dilution; Cell Signaling Technology), TXNIP (1:2000 dilution; Cell Signaling Technology), nuclear factor kappa B (NF-κB) p65 (1:500 dilution; Cell Signaling Technology), p-NF-κB p65 (1:2000 dilution; Cell Signaling Technology), Histone H3 (1:5000 dilution; Gene Tex, San Antonio, TX, USA) and GAPDH (1:1000 dilution; Proteintech Group, Rosemont, IL, USA) overnight at 4°C. After washing by TBST buffer, the membranes were incubated with secondary antibodies for goat anti-rabbit or goat anti-mouse IgG-HRP (1:3000 dilution; Solarbio Science & Technology) for 1 h at 37°C. The membranes were then treated with an enhanced chemiluminescence substrate (Solarbio Science & Technology), exposed in darkness and scanned for analysis of optical density values using Gel-Pro-Analyzer software.

### Quantification of TNF-α, IL-1β, DHEA, and testosterone

The level of TNF-α in ovarian tissues was determined using a mouse TNF-α high-sensitivity ELISA kit (MultiSciences Biotech, Hangzhou, China). The level of IL-1β in ovarian tissues and the culture supernatant of KGN cells were analyzed by a mouse or a human IL-1β high-sensitivity ELISA kit (MultiSciences Biotech). The levels of DHEA and testosterone in the culture supernatant of KGN cells were quantified by a DHEA ELISA kit (USCN Life Science) and a testosterone ELISA kit (USCN Life Science), respectively.

### Cell culture and transfection

KGN cells (Procell Life Science & Technology, Wuhan, China) were incubated in a mixture of Dulbecco’s modified Eagle medium and Ham’s F-12 medium (DMEM/F12, Procell Life Science & Technology) containing 10% fetal bovine serum (Hyclone, South Logan, UT, USA). KGN cells used in this study were authenticated by short tandem repeat (STR) analysis and identified as mycoplasma-free cells using Mycoplasma PCR Detection Kit (Wanleibio, Shenyang, China) (supplementary Figure 1). Here, we used TNF-α-induced KGN cells as an *in vitro* research model to mimic PCOS-related cell injury, which is in line with previous studies [33–35]. Cells were seeded in 6-well plates at a density of 1 × 10^5^ and cultured in an incubator with 5% CO_2_ at 37°C. For TNF-α treatment, cells were incubated in a serum-free medium for 24 h and then treated with 100 ng/ml TNF-α for 24 h. Lipofectamine 2000 (Invitrogen, Carlsbad, CA, USA) was used for transfection according to manufacturer’s instructions [29,36]. KGN cells were transfected with 100 pmol of miR-1224-5p mimics or negative control (NC) mimics or co-transfected with 50 pmol of miR-1224-5p mimics and 1 μg of FOXO1 overexpressing plasmid (pcDNA3.1-FOXO1) or empty plasmid (pcDNA3.1-vector). After 24-h transfection, the cells were subjected to TNF-α treatment as described above.

### Quantification of caspase-1 activity

KGN cells were incubated with a lysis buffer for 15 min to extract the protein. The concentration of extracted protein was quantified by a Bradford protein assay kit (Beyotime Institute of Biotechnology, Haimen, China). Then, the activity of caspase-1 was determined by a caspase-1 activity assay kit (Beyotime Institute of Biotechnology) following the manufacturer’s instruction [37].

### Immunofluorescence staining for NF-κB p65

Immunofluorescence staining was performed to detect the nuclear translocation of NF-κB p65 in KGN cells [38]. KGN cells grown on coverslips were fixed in 4% paraformaldehyde (Sinopharm Chemical Reagent, Beijing, China) for 15 min, incubated with 0.1% Triton X-100 (Beyotime Institute of Biotechnology) for 30 min and washed by phosphate buffer (PBS) for 3 times. Then, the cells were immersed in goat serum (Solarbio Science & Technology) at room temperature for 30 min and incubated with primary NF-κB p65 antibody (1:200, Proteintech Group) at 4°C overnight followed by secondary Cy3-labeled goat anti-rabbit IgG antibody (1:200, Beyotime Institute of Biotechnology) at room temperature for 1 h. After rinsing in PBS for 3 times, the cells were stained with 4ʹ,6-diamidino-2-phenylindole (DAPI, Beyotime Institute of Biotechnology), covered with mounting medium (Solarbio Science & Technology) and observed under a fluorescence microscope at a magnification of 400 × .

### Luciferase reporter assay

According to the prediction results, wild-type (Wt) FOXO1 3ʹUTR or mutant (Mut) FOXO1 3ʹUTR sequence targeted by miR-1224-5p was synthesized and subjected to a luciferase reporter assay [39]. HEK-293 T cells (Zhong Qiao Xin Zhou Biotechnology, Shanghai, China) were incubated in DMEM supplemented with 10% FBS at 37°C. Cells were seeded in 12-well plates at a density of 5 × 10^4^ and cultured in an incubator with 5% CO_2_ at 37°C. Before transfection, 293 T cells were incubated in a serum-free medium for 1 h. The cells were co-transfected with miR-1224-5p mimics or NC mimics and luciferase reporter plasmid containing wild-type (Wt) FOXO1 3ʹUTR or mutant (Mut) FOXO1 3ʹUTR using Lipofectamine 2000. After transfection for 48 h, 293 T cells were collected to detect firefly and Renilla luciferase activity using a Dual luciferase reporter gene assay kit (KeyGen Biotech, Nanjing, China).

### Statistical analysis

Each *in vivo* experiment was biologically repeated for 6 times (n = 6), and *in vitro* experiment was conducted with three biological repetitions. All data were shown as mean ± standard deviation (SD). Statistical analysis for comparing two groups was performed by a student’s *t*-test. For comparing three or more groups, the statistical analysis was performed by one-way analysis of variance followed by Tukey’s post hoc test (GraphPad Prism 8.0). A P < 0.05 was regarded as statistically significant.

## Results

### Downregulation of miR-1224-5p was accompanied with enhanced activation of NLRP3 inflammasome in dehydroepiandrosterone (DHEA)-induced PCOS mice

**Figure 1.**
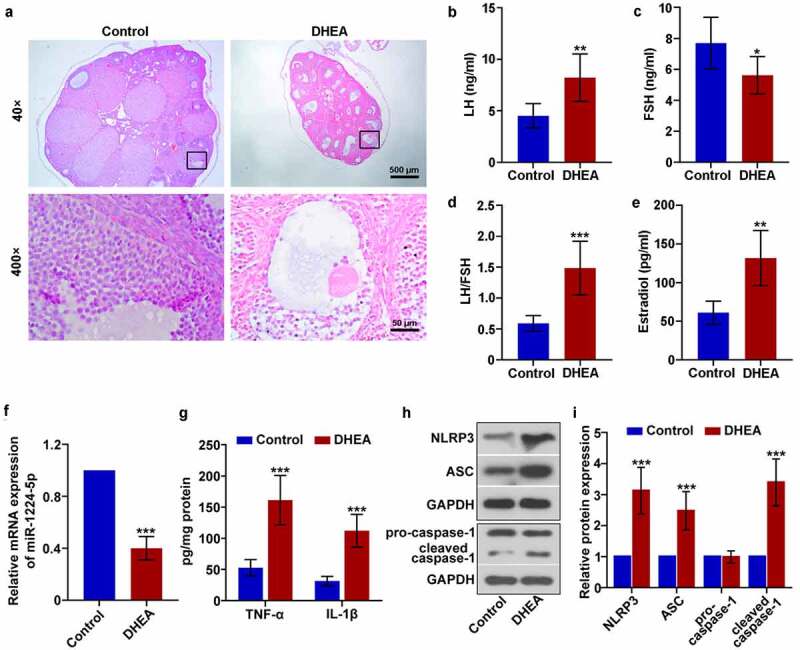
Downregulation of miR-1224-5p was accompanied with enhanced activation of NLRP3 inflammasome in dehydroepiandrosterone (DHEA)-induced polycystic ovary syndrome (PCOS) mice. (a) Representative images of the ovarian tissue after H&E staining. The levels of (b) luteinizing hormone (LH) and (c) follicle-stimulating hormone (FSH) in the serum of mice. (d) The ratio of LH to FSH. (e) The level of estradiol in the serum of mice. (f) The relative mRNA expression of miR-1224-5p in the ovarian tissue of mice quantified by real-time PCR. (g) The levels of tumor necrosis factor (TNF)-α and interleukin (IL)-1β assessed by ELISA kits. (h) The protein bands and (i) relative protein expression of NOD-like receptor protein 3 (NLRP3), apoptosis-associated speck-like protein containing a C-terminal caspase recruitment domain (ASC), pro-caspase-1 and cleaved caspase-1 quantified by western blotting analysis. Data were shown as mean ± SD (n = 6). *p < 0.05, **p < 0.01, ***p < 0.001 versus Control group

To investigate the expression and function of miR-1224-5p in PCOS *in vivo*, we established a mouse model of PCOS model by daily subcutaneous injection of DHEA (60 mg/kg) for 3 weeks. Three weeks later, mice treated with DHEA showed significantly enhanced fast blood glucose level, fast insulin level and HOMA-IR index, compared to Control group (Table 2). H&E staining was then performed to assess histological changes in the ovarian tissue of the mice. It was observed that mice in DHEA group showed increased cystic follicles and decreased corpus luteum in the ovarian tissue, while mice in Control group exhibited normal ovarian morphology (Figure 1(a)). Moreover, the granulosa cells in DHEA group were loosely arranged as presented in the images at higher magnification (400×). We also determined the levels of LH, FSH and estradiol in the serum of mice. It was found that the serum levels of LH (Figure 1(b)) and estradiol (Figure 1(e)) were significantly enhanced in mice treated with DHEA. A lower serum level of FSH was showed in DHEA mice than controls (Figure 1(c)). Accordingly, the ratio of LH/FSH was dramatically increased in mice of DHEA group (Figure 1(d)). Therefore, we demonstrated that DHEA successfully induced the features of PCOS in mice in the present study. The mRNA expression of miR-1224-5p in ovarian tissue of mice was then determined by real-time PCR. It was found that miR-1224-5p expression in mouse ovarian tissue was significantly downregulated by DHEA (figure 1(f)). Moreover, treatment with DHEA remarkably elevated the levels of proinflammatory factors TNF-α and IL-1β in the ovarian tissue of mice (Figure 1(g)). The activation of NLRP3 inflammasome in ovarian tissue was assessed by western blotting assay. The relative expression of NLRP3, ASC and cleaved caspase-1 was dramatically upregulated in DHEA group, demonstrating that DHEA induced the activation of NLRP3 inflammasome in the ovarian tissue of mice (Figure 1(h-i)). The expression of pro-caspase-1 had no statistical difference between Control group and DHEA group.

**Table 2.** Fast glucose level, fast insulin level and HOMA-IR index of mice

	Control (n = 6)	DHEA (n = 6)	P values
Fast glucose level (mmol/L)	4.64 ± 0.38	7.28 ± 0.26	<0.001
Fast insulin level (mIU/L)	9.14 ± 1.31	20.17 ± 3.79	<0.001
HOMA-IR index	1.90 ± 0.39	6.54 ± 1.35	<0.001

### Upregulation of miR-1224-5p attenuated TNF-α-induced secretion of androgens and inflammation in KGN cells

**Figure 2.**
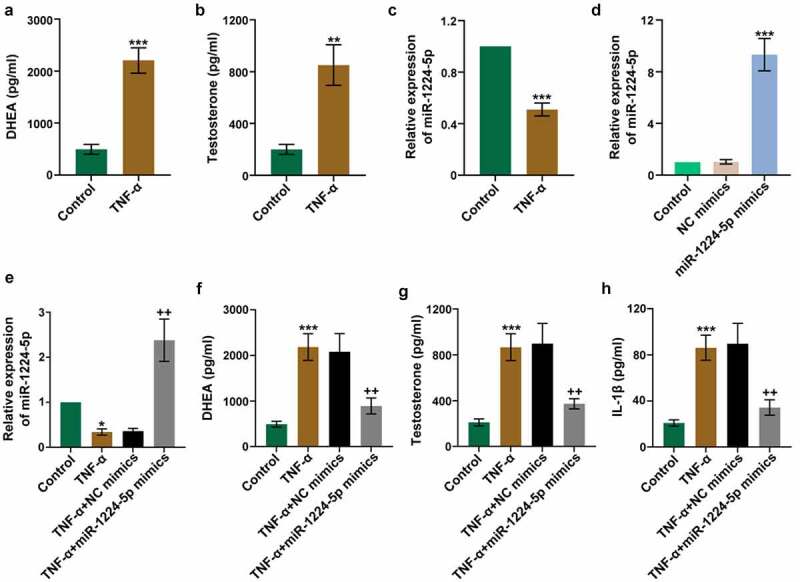
Upregulation of miR-1224-5p attenuated TNF-α-induced secretion of androgens and inflammation in human granulosa-like tumor (KGN) cells. KGN cells were treated with TNF-α for 24 h to establish an *in vitro* study model. The levels of (a) DHEA and (b) testosterone in TNF-α-treated KGN cells versus controls. (c) The relative mRNA expression of miR-1224-5p following TNF-α treatment. To investigate the role of miR-1224-5p, KGN cells were transfected with miR-1224-5p mimics or negative control (NC) mimics, followed by TNF-α treatment. (d) The relative mRNA expression of miR-1224-5p was determined to verify the efficiency of transfection. (e) The relative mRNA expression of miR-1224-5p in KGN cells with different treatments. The levels of (f) DHEA, (g) testosterone and (h) IL-1β in KGN cells with different treatments. Data were shown as mean ± SD (n = 3). *p < 0.05, **p < 0.01, ***p < 0.001 versus Control group. ++p < 0.01, versus TNF-α+ NC mimics group

The role of miR-1224-5p was further investigated in TNF-α-induced KGN cells, an *in vitro* research model to mimic PCOS-related cell injury. We found that TNF-α significantly enhanced the levels of DHEA and testosterone in KGN cells, demonstrating that TNF-α treatment induced the secretion of androgens *in vitro* (Figure 2(a-b)). Moreover, TNF-α induction greatly downregulated the expression of miR-1224-5p in KGN cells (Figure 2(c)). To investigate the role of miR-1224-5p *in vitro*, KGN cells were transfected with miR-1224-5p mimics. Real-time PCR analysis was performed to assess the efficiency of transfection (Figure 2(d)). The results showed that miR-1224-5p mimics efficiently upregulated the expression of miR-1224-5p in KGN cells in the presence of TNF-α or not (Figure 2(d-e)). Further, the levels of DHEA and testosterone in KGN cells were significantly decreased by upregulation of miR-1224-5p, indicating the inhibitory effect of miR-1224-5p on TNF-α-induced secretion of androgens (figure 2(f-g)). The level of TNF-α-induced IL-1β was also dramatically reduced by the enforced expression of miR-1224-5p (Figure 2(h)). These results suggested that upregulation of miR-1224-5p attenuated TNF-α-induced inflammation in KGN cells.

### Upregulation of miR-1224-5p inhibited the activation of NLRP3 inflammasome in KGN cells

**Figure 3.**
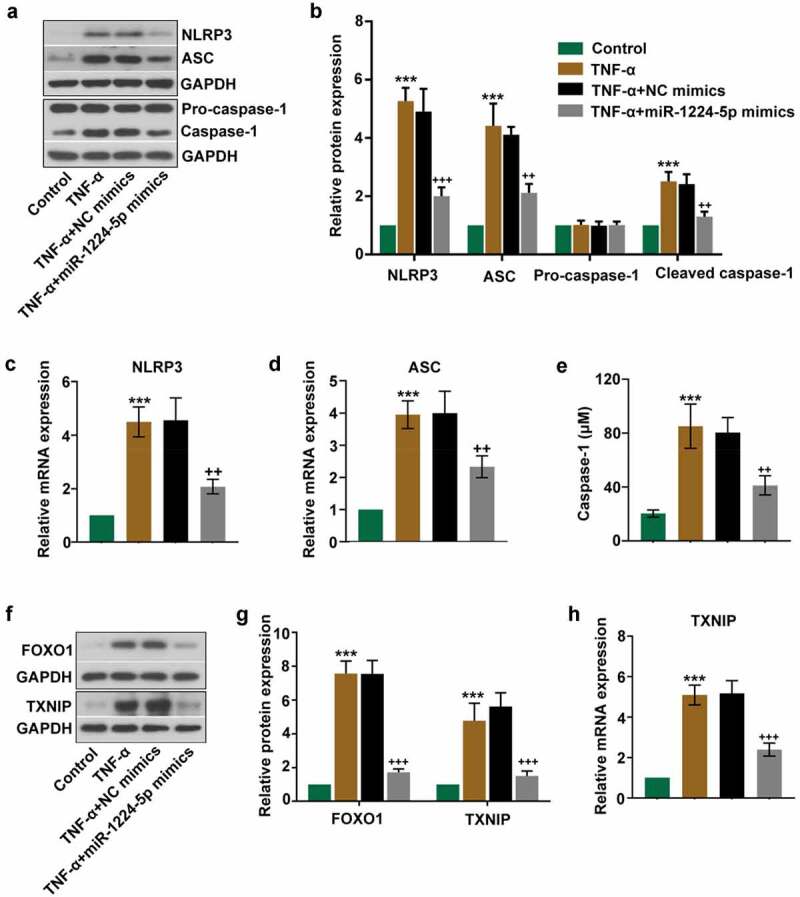
Upregulation of miR-1224-5p inhibited the activation of NLRP3 inflammasome in TNF-α-treated KGN cells. miR-1224-5p was enforced expressed in KGN cells with miR-1224-5p mimics. After 24-h transfection, cells were induced with TNF-α for 24 h. (a) The protein bands and (b) the relative protein expression of NLRP3, ASC, pro-caspase-1 and cleaved caspase-1 in KGN cells. The relative mRNA expression of (c) NLRP3 and (d) ASC in KGN cells. (e) The level of caspase-1 in KGN cells quantified by the ELISA kit. (f) The protein bands and (g) the relative protein expression of FOXO1 and thioredoxin interaction protein (TXNIP) in KGN cells. (h) The relative mRNA expression of TXNIP in KGN cells. Data were shown as mean ± SD (n = 3). ***p < 0.001, versus Control group. ++p < 0.01, +++p < 0.001 versus TNF-α+ NC mimics group

We next evaluated the effects of miR-1224-5p on NLRP3 inflammasome activation by assessing the associated proteins in TNF-α-treated KGN cells. The mRNA and protein expression of NLRP3 and ASC were dramatically upregulated by TNF-α treatment (Figure 3(a-d)). The protein expression of cleaved caspase-1 and the activity of caspase-1 were also significantly enhanced in TNF-α group (Figure 3(a-b, e)). These results indicated that TNF-α induced the activation of NLRP3 inflammasome in KGN cells. However, TNF-α-induced expression of NLRP3, ASC and cleaved caspase-1 and the activity of caspase-1 were greatly counteracted by the upregulation of miR-1224-5p (Figure 3(a-e)), suggesting that miR-1224-5p inhibited TNF-α-induced activation of NLRP3 inflammasome in KGN cells. The relative expression of pro-caspase-1 had no statistical difference among these groups (Figure 3a-b). Furthermore, we determined the expression of FOXO1 and TXNIP in KGN cells following TNF-α treatment. The protein and/or mRNA expression of FOXO1 and TXNIP was strongly upregulated by TNF-α treatment, while downregulated by transfecting with miR-1224-5p mimics (figure 3(f-h)), indicating that miR-1224-5p might be a negative regulator of FOXO1 and TXNIP expression.

### Upregulation of miR-1224-5p inhibited the activation of NF-κB p65 in KGN cells

**Figure 4.**
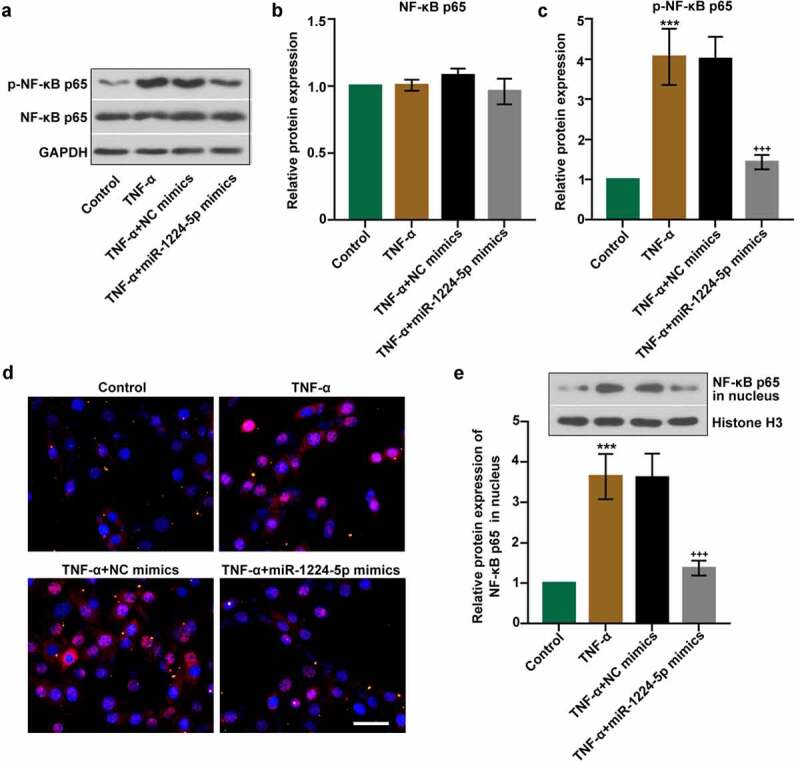
Upregulation of miR-1224-5p inhibited the activation of nuclear factor kappa B (NF-κB) p65 in KGN cells. (a) The protein bands and the relative protein expression of (b) NF-κB p65 and (c) p-NF-κB p65 in KGN cells. (d) Representative images of immunofluorescence staining for NF-κB p65. Scale bar = 50 μm. (e) The protein bands and the relative protein expression of NF-κB p65 in the nucleus in KGN cells. Data were shown as mean ± SD (n = 3). ***p < 0.001, versus Control group. +++p < 0.001, versus TNF-α+ NC mimics group

The involvement of NF-κB signaling pathway was examined to further unveil the underlying mechanism of miR-1224-5p. The expression of p-NF-κB p65 and NF-κB p65 was determined in TNF-α-treated KGN cells with or without miR-1224-5p overexpression by Western blotting assay. We found no statistical difference in the expression of NF-κB p65 among four groups (Figure 4(a-b)). The expression of p-NF-κB p65 was noticeably elevated in TNF-α group, implicating that TNF-α induced that activation of NF-κB p65 in KGN cells (Figure 4(c)). In comparison, the expression of p-NF-κB p65 was significantly downregulated by upregulation of miR-1224-5p, implying that miR-1224-5p inhibited the activation of NF-κB p65 in KGN cells. We further performed immunofluorescence staining for NF-κB p65 in KGN cells (Figure 4(d)). Treatment with TNF-α enhanced the translocation of NF-κB p65 into the nucleus of KGN cells, implying TNF-α-induced NF-κB p65 activation *in vitro*. Reduced activation of NF-κB p65 was observed KGN cells with miR-1224-5p overexpression. Besides, western blotting analysis showed a similar pattern of changes in the protein expression of NF-κB p65 in nucleus (Figure 4(e)). Upregulation of miR-1224-5p decreased the translocation of NF-κB p65 into nucleus, suggesting the suppression of miR-1224-5p in TNF-α-induced activation of NF-κB p65 in KGN cells.

### MiR-1224-5p inhibited the activation of NLRP3 inflammasome in KGN cells by targeting FOXO1

**Figure 5.**
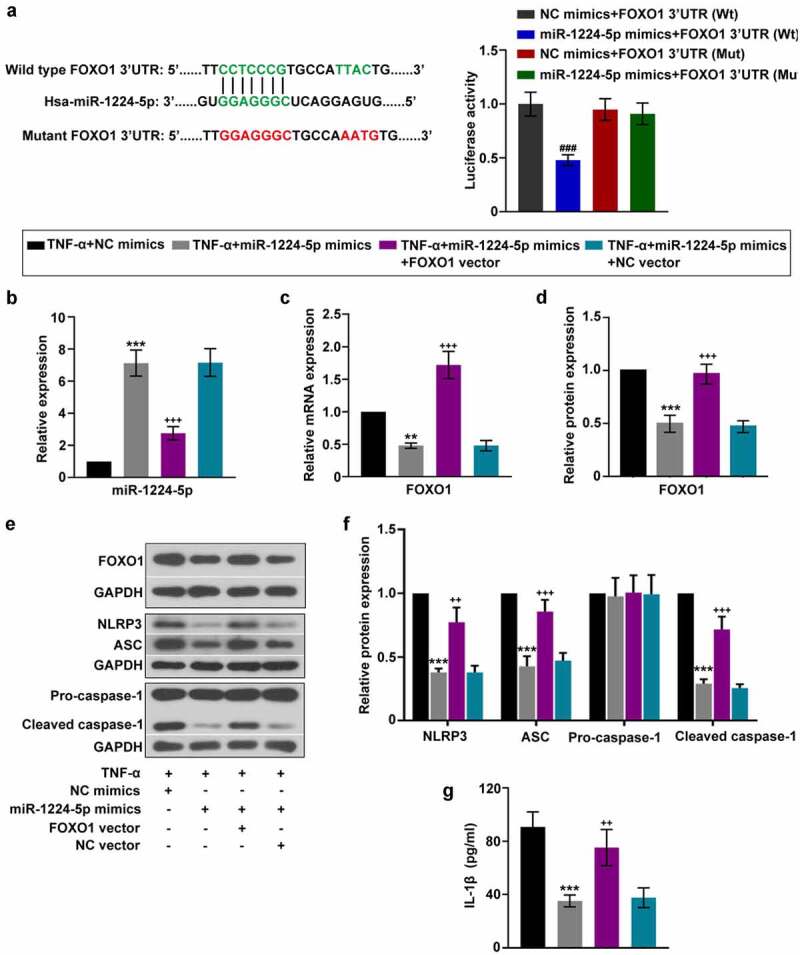
miR-1224-5p inhibited the activation of NLRP3 inflammasome in KGN cells by targeting FOXO1. To investigate the involvement of FOXO1 in the regulation of miR-1224-5p, FOXO1 was enforced upregulated in KGN cells with miR-1224-5p overexpression. (a) Predicted binding site of miR-1224-5p in wild-type FOXO1 3ʹUTR and mutant sites. Luciferase reporter assay was performed in HEK-293 T cells. Following different treatment, (b) the relative mRNA expression of miR-1224-5p in KGN cells. (c-d) The relative mRNA and protein expression of FOXO1 in KGN cells. (e) The protein band and (f) the relative protein expression of NLRP3, ASC, pro-caspase-1 and cleaved caspase-1 in KGN cells. (g) ELISA analysis of IL-1β level in KGN cells. Data were shown as mean ± SD (n = 3). ###P < 0.001, versus NC mimics+FOXO1 3ʹUTR (Wt) group or miR-1224-5p mimics+FOXO1 3ʹUTR (Mut) group. **p < 0.01, ***p < 0.001 versus TNF-α+ NC mimics group. ++p < 0.01, +++p < 0.001 versus TNF-α+ miR-1224-5p mimics+NC vector group

It is predicted that FOXO1 is a potential target gene of miR-1224-5p. The relationship between miR-1224-5p and FOXO1 was further verified by luciferase reporter assay (Figure 5(a)). HEK-293 T cells co-transfected with miR-1224-5p mimics and plasmid containing Wt-FOXO1 3ʹUTR showed lower luciferase activity than that with miR-1224-5p mimics and plasmid containing Mut-FOXO1 3ʹUTR. Besides, there is no significant difference in the luciferase activity between cells transfected with NC mimics and miR-1224-5p mimics in Mut-FOXO1 3ʹUTR system. The mRNA and protein expression of FOXO1 were significantly downregulated by overexpression of miR-1224-5p in KGN cells in the presence of TNF-α (Figure 5(b-d)). These results indicated that miR-1224-5p directly targets FOXO1 3ʹUTR and suppressed FOXO1 expression. Thus, we demonstrated that FOXO1 is a target of miR-1224-5p. To investigate whether miR-1224-5p inhibited the activation of NLRP3 inflammasome through regulating FOXO1, FOXO1 was enforced to be upregulated in KGN cells with miR-1224-5p overexpression. The mRNA and protein exprsssion of FOXO1 was significantly elevated in KGN cells after transfection with FOXO1 overexpressing plasmid, confirming high efficiency of the transfection (Figure 5(c-d)). The expression of miR-1224-5p was negatively correlated with FOXO1 in KGN cells (Figure 5(b-d)). In addition, the inhibitory effects of miR-1224-5p on NLRP3, ASC and cleaved caspase-1 expression were largely diminished by FOXO1 overexpression, indicating that miR-1224-5p might function to inhibit the activation of NLRP3 inflammasome through downregulating FOXO1 expression in KGN cells (Figure 5(e-f)). Similarly, miR-1224-5p-induced downregulation of IL-1β was reverted by further overexpression of FOXO1 in TNF-α-treated cells (Figure 5(g)). Thus, we suggested miR-1224-5p/FOXO1 as potential mechanism involved in the regulation of the activation of NLRP3 inflammasome in KGN cells.

## Discussion

PCOS poses a great threat to women of reproductive age, which may result in hyperandrogenism, oligo-anovulation, and even anovulatory infertility [2]. The development of PCOS is closely associated with multiple endogenous and exogenous factors, such as genetics, insulin resistance and obesity [40,41]. Recently, an increasing number of miRNAs have been identified to be aberrantly expressed in the follicular fluid of women with PCOS, implying the possible effects of these miRNAs on regulating the progression of PCOS [22,42,43]. Therefore, it is important to elucidate the specific roles of these aberrantly expressed miRNAs, which may provide novel therapeutic targets for PCOS. MiR-1224-5p is one of them, and it has been reported to be significantly downregulated in the follicular fluid of patients with PCOS [23]. Nevertheless, it is unclear whether miR-1224-5p plays a critical role in mediating the development of PCOS. In this study, we investigated the potential effect of miR-1224-5p in a mouse model of PCOS and TNF-α-treated KGN cells.

The mouse model of PCOS was constructed by daily injection of DHEA continuously for 3 weeks. DHEA is a commonly used androgen to induce some typical features *in vivo* which are similar to human PCOS, such as hyperandrogenism, anovulation and insulin resistance [44]. In the present study, DHEA-induced mice showed dramatically enhanced fast glucose level, fast insulin level and HOMA-IR index, indicating that DHEA treatment caused insulin resistance in the mice. Moreover, treatment with DHEA induced several key features of PCOS in the mice, including polycystic ovary, increased LH level and LH/FSH ratio. These results demonstrated that the PCOS model was successfully established in mice. Importantly, we found that the relative expression of miR-1224-5p was greatly downregulated in the ovarian tissue of PCOS mice compared to normal mice. Moreover, the levels of TNF-α and IL-1β were dramatically elevated in PCOS mice, indicating the aggravated inflammation induced by DHEA. Previous researches have highlighted that chronic low-grade inflammation contributes enormously to the pathogenesis of PCOS [45,46]. TNF-α is a well-known proinflammatory cytokine, which functions as an important mediator of insulin resistance in PCOS. The function of TNF-α in insulin resistance is realized by serine phosphorylation of insulin receptor substrate-1 and downregulation of insulin-sensitive glucose transport protein [47]. Circulating level of TNF-α is found to be enhanced in women with PCOS, as well as in animal models of PCOS. Consistent with previous studies, we observed a dramatically elevated level of TNF-α in PCOS mice as well.

More noticeably, we detected the activation of NLRP3 inflammasome in DHEA-induced PCOS mice as evidenced by increased expression of the components of NLRP3 inflammasome, including NLRP3, ASC and cleaved caspase-1, and subsequent production of IL-1β. NLRP3 inflammasome is an intracellular signaling platform comprised of NLRP3, ASC and caspase-1, which plays a critical role in the host immune response. Activation of NLRP3 inflammasome induces the secretion of proinflammatory IL-1β and IL-18, further recruiting the immune cells to the infected site [48]. The pathology of many human disorders involves the participation of activated NLRP3 inflammasome [49,50]. However, the role of NLRP3 inflammasome in PCOS remains largely unknown. We found a downregulated expression of miR-1224-5p and upregulated activation of NLRP3 inflammasome in the ovarian tissue of PCOS mice, indicating the potential importance of miR-1224-5p in regulating the activation of NLRP3 inflammasome induced by DHEA *in vitro*. To explore whether miR-1224-5p involves in the activation of NLRP3 inflammasome and the underlying mechanism, more study was then carried out on KGN cells.

Previous studies have demonstrated that TNF-α affects the secretion of androgen in KGN cells [51]. Therefore, in the present study, exogenous TNF-α was used to induce the pathological changes in KGN cells. As expected, treating with TNF-α significantly enhanced the levels of DHEA and testosterone in KGN cells. Meanwhile, the relative expression of miR-1224-5p was greatly inhibited by TNF-α treatment. Further study showed that upregulation of miR-1224-5p could remarkably reduce the levels of DHEA and testosterone, as well as the level of proinflammatory IL-1β. These results indicated the effect of miR-1224-5p on alleviating TNF-α-induced androgen excess and inflammation in KGN cells. It has been reported that hyperandrogenism and inflammation interact mutually in the pathology of PCOS [52]. Circulating biomarkers of inflammation are closely influenced by circulating androgens. By contrast, the production of androgens in ovarian may be promoted by local inflammatory response. Our research confirmed that the production of androgen in KGN cells could be induced by TNF-α. Moreover, we found that TNF-α induced activation of NLRP3 inflammasome, which was inhibited by upregulation of miR-1224-5p. Therefore, we speculated that the effect of miR-1224-5p on attenuating TNF-α-induced androgen and inflammation in KGN cells might be achieved by inhibiting the activation of NLRP3 inflammasome.

More interestingly, the relative expression of FOXO1 and TXNIP was found to be dramatically increased by TNF-α treatment in KGN cells. FOXO1 is a common transcriptional factor that regulates multiple cellular metabolisms [53]. TXNIP primarily functions to inhibit thioredoxin (an important redox protein) and induces the production of intracellular ROS, further promoting the progression of inflammation [54]. Moreover, TXNIP has been recognized as a novel mediator in the activation of NLRP3 inflammasome, which is involved in the molecular mechanism of a wide range of diseases [55–57]. FOXO1 is reported to positively regulate the expression of TXNIP and contribute to the production of ROS [58]. The abnormally elevated expression of TXNIP has been determined in patients with PCOS [17]. Therefore, it could be speculated that FOXO1 and TXNIP might participate in the activation of NLRP3 inflammasome in the present research.

NF-κB family plays an important role in mediating the inflammatory response in various cell types, which drives the recruitment of inflammatory cells and the release of inflammatory cytokines through regulating target genes [59]. NF-κB p65 is a member of the NF-κB family. When inactivated, NF-κB p65 combines with the inhibitor of κB (IκB) and locates in the cytoplasm [60]. However, NF-κB p65 activates and translocates into the nucleus to realize its function in transcriptional regulation under specific stimulations. In this study, we found that TNF-α treatment resulted in the translocation of NF-κB p65 from cytoplasm to the nucleus in KGN cells. In addition, the protein expression of NF-κB p65 in nucleus was significantly upregulated in TNF-α-treated cells as well. These findings indicated that TNF-α induced the activation of NF-κB p65 in KGN cells. Notably, the upregulation of miR-1224-5p inhibited the activation of NF-κB p65, revealing that inhibiting the activation of NF-κB p65 might be involved in the regulation of miR-1224-5p in attenuating TNF-α induced inflammation in KGN cells. We also found that the upregulation of miR-1224-5p greatly inhibited the expression of FOXO1 and TXNIP. Luciferase reporter assay verified that FOXO1 is directly targeted by miR-1224-5p. To investigate whether miR-1224-5p inhibited the activation of NLRP3 inflammasome by targeting FOXO1, we upregulated the expression of FOXO1 in KGN cells and studied the activation of NLRP3 inflammasome. It was found that the upregulation of FOXO1 resulted in downregulation of miR-1224-5p and upregulation of NLRP3 inflammasome-asspcoated proteins as well as IL-1β production, indicating the regulation of miR-1224-5p/FOXO1 in the activation of NLRP3 inflammasome. Thus, we demonstrated that miR-1224-5p might inhibit the activation of NLRP3 inflammasome by targeting FOXO1 in KGN cells.

Notably, FOXO1 has been reported to regulate insulin action in multiple metabolic processes [61,62]. Moreover, FOXO1 could promote the production of IL-1β in macrophages and contribute to insulin resistance [63]. Inhibition of FOXO1 improved fast glycemia and hepatic glucose production in diabetic mice [64]. These findings suggest the potential role of FOXO1 in promoting insulin resistance. However, little is known about the relationship between FOXO1 and insulin resistance in PCOS. Insulin resistance is a common feature of PCOS. The pathogenesis of insulin resistance is closely associated with the chronic inflammation. It is well known that the insulin signaling transduction can be blocked by several proinflammatory cytokines, including TNF-α and IL-6 [65]. In the present study, miR-1224-5p was found to exert an anti-inflammatory role in TNF-α-treated KGN cells by suppressing the activation of NLRP3 inflammasome and NF-κB signaling pathway. Besides, miR-1224-5p functioned in KGN cells through negatively regulating the expression of FOXO1. Therefore, we speculated that miR-1224-5p might play a role in the regulation of insulin resistance in PCOS. However, more research should be carried out to investigate the role of miR-1224-5p and FOXO1 in the insulin resistance in PCOS.

## Conclusion

**Figure 6.**
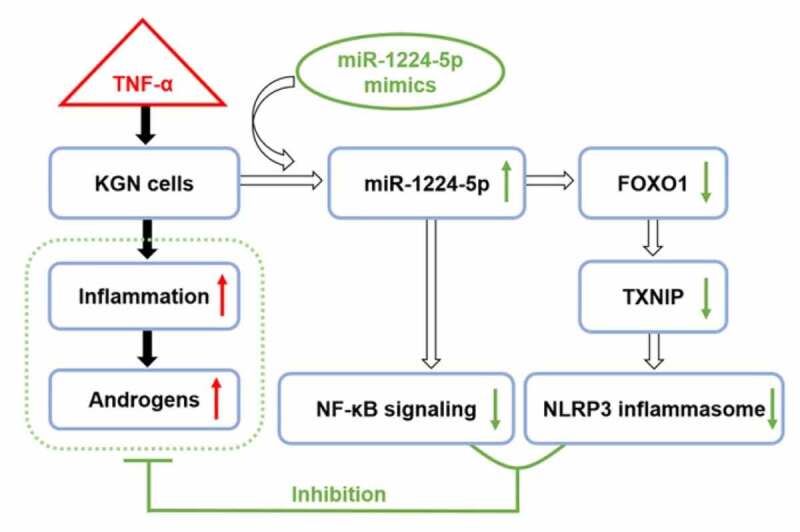
The possible mechanism underlying the regulation of miR-1224-5p/FOXO1 in attenuating the inflammation and androgen excess in TNF-α-treated KGN cells

In conclusion, miR-1224-5p was found to be downregulated in DHEA-induced PCOS mice. *In vitro* study showed that miR-1224-5p functioned to attenuate TNF-α-induced inflammation through inhibiting the activation of NLRP3 inflammasome and NF-κB p65 signaling in KGN cells by targeting FOXO1 (Figure 6). Therefore, our study demonstrated the regulation of miR-1224-5p/FOXO1 in KGN cells, which provides insights into a potential mechanism in the pathogenesis of PCOS and a promising target for treating PCOS.

## Supplementary Material

Supplemental Material

## Data Availability

The data of this study are available from the corresponding author upon reasonable request.

## Highlights

- The expression of miR-1224-5p was decreased in DHEA-induced mouse model of PCOS.
- MiR-1224-5p inhibited NLRP3 inflammasome activation in TNF-α-induced KGN cells.
- MiR-1224-5p suppressed NF-κB signaling in TNF-α-induced KGN cells.
- MiR-1224-5p functioned through directly targeting FOXO1.
