# Supplementary figures and images for: MiR-1224-5p attenuates polycystic ovary syndrome through inhibiting NOD-like receptor protein 3 inflammasome activation via targeting Forkhead box O 1
Source: Bioengineered. 2021 Oct 21;12(1):8555–69. doi: 10.1080/21655979.2021.1987125 (PMC8806973; doi:10.1080/21655979.2021.1987125)

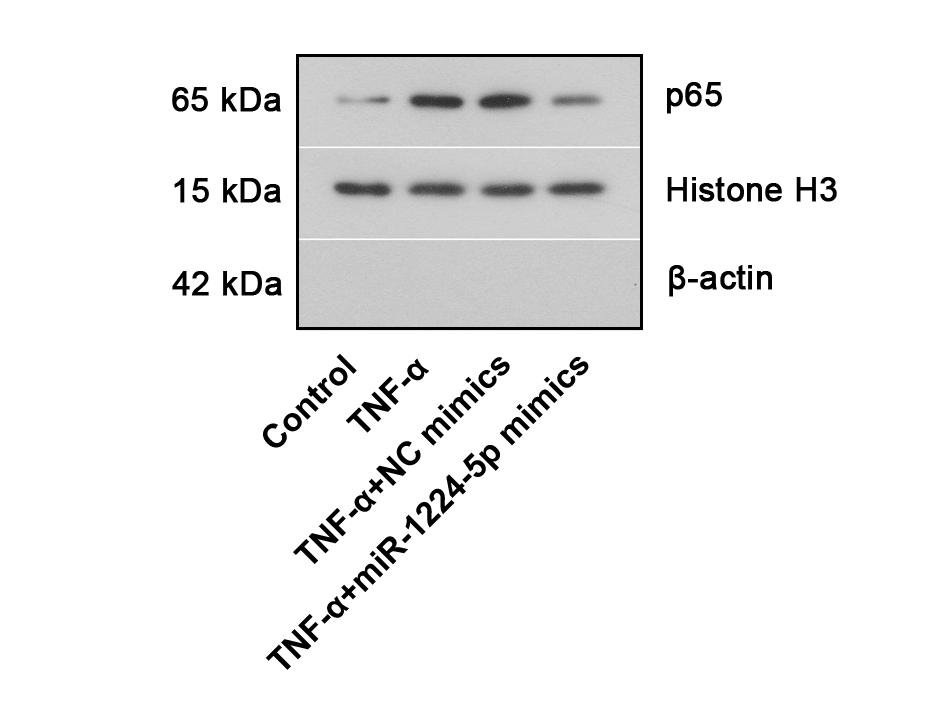

Supplement: Supplemental Material [file KBIE_A_1987125_SM2852.tif]
